# A renewed tool kit to explore *Chlamydia* pathogenesis: from molecular genetics to new infection models

**DOI:** 10.12688/f1000research.18832.1

**Published:** 2019-06-21

**Authors:** Lee Dolat, Raphael H Valdivia

**Affiliations:** 1 Department of Molecular Genetics and Microbiology, Duke University Medical Center, Durham, USA

**Keywords:** Chlamydia, pathogenesis, genetics, cell-cell junctions, organoids, infection models

## Abstract

*Chlamydia trachomatis* is the most prevalent sexually transmitted bacterial pathogen and the leading cause of preventable blindness in the developing world. *C. trachomatis* invades the epithelium of the conjunctiva and genital tract and replicates within an intracellular membrane-bound compartment termed the inclusion. To invade and replicate in mammalian cells, *Chlamydia* remodels epithelial surfaces by reorganizing the cytoskeleton and cell–cell adhesions, reprograms membrane trafficking, and modulates cell signaling to dampen innate immune responses. If the infection ascends to the upper female genital tract, it can result in pelvic inflammatory disease and tissue scarring. *C. trachomatis* infections are associated with infertility, ectopic pregnancies, the fibrotic disorder endometriosis, and potentially cancers of the cervix and uterus. Unfortunately, the molecular mechanisms by which this clinically important human pathogen subverts host cellular functions and causes disease have remained relatively poorly understood because of the dearth of molecular genetic tools to study *Chlamydiae* and limitations of both *in vivo* and *in vitro* infection models. In this review, we discuss recent advances in the experimental molecular tool kit available to dissect *C. trachomatis* infections with a special focus on *Chlamydia*-induced epithelial barrier disruption by regulating the structure, function, and dynamics of epithelial cell–cell junctions.

## Introduction

The order *Chlamydiae* are obligate intracellular pathogens of eukaryotic cells. These bacteria have reduced genomes and display biphasic developmental stages that alternate between distinct extracellular and intracellular forms ^1, 2^. Eleven pathogenic *Chlamydia* species infect vertebrate animals and display tissue-specific tropism ^3^. The elementary body (EB) is the environmentally stable form of the pathogen that binds and invades target cells. The EB then transitions into the larger intracellular reticulate body (RB) form. RBs replicate and secrete proteins across the parasitophorous membrane-bound vacuole (“inclusion”) to modulate multiple host cellular functions that benefit the bacterium ^4^.

**Figure 1.**
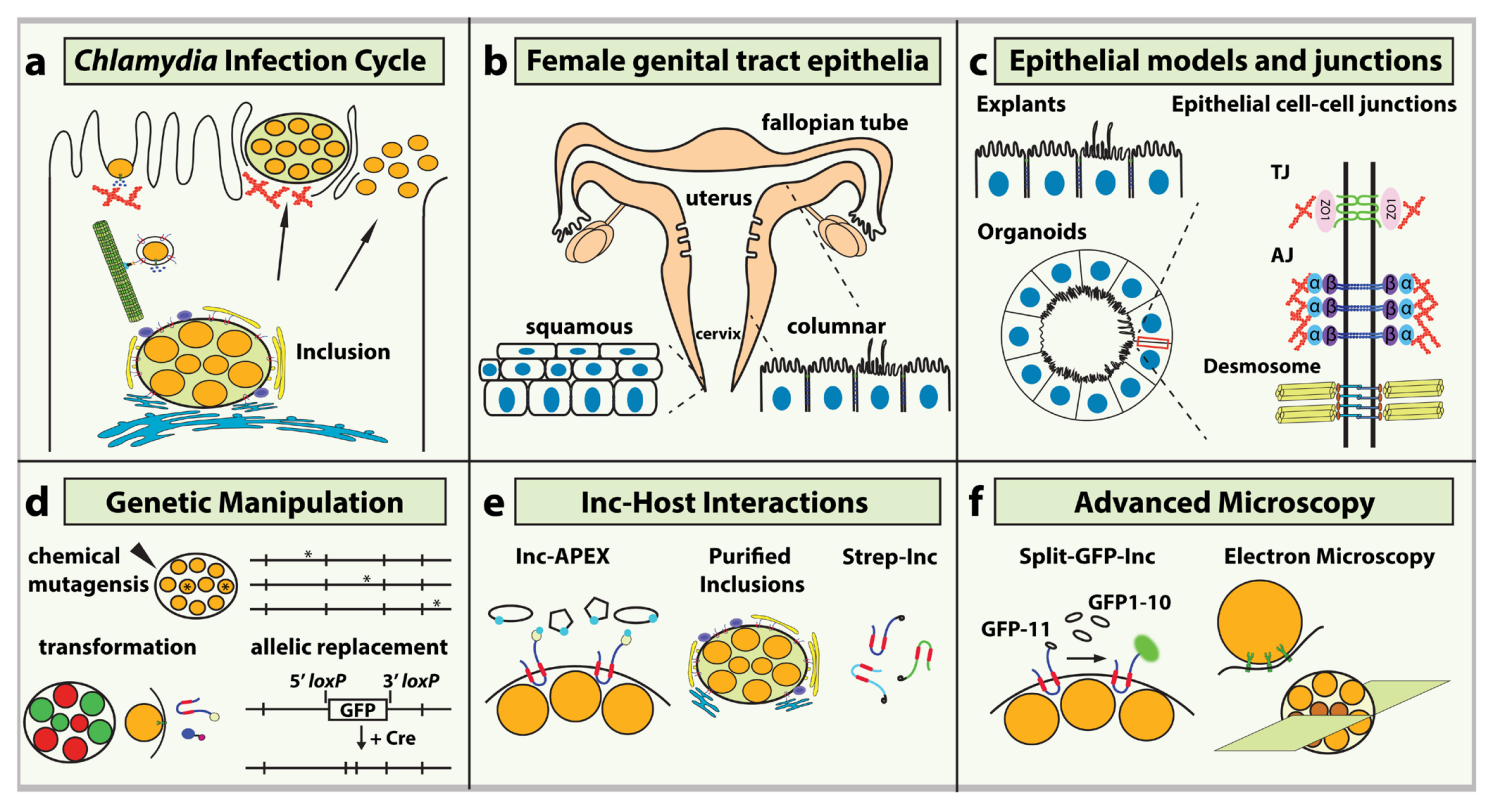
Recent advances in the molecular tool kit and infection models to explore *Chlamydia* infection. ( **a**) The *Chlamydia trachomatis* infection cycle. The elementary body (EB) form of the bacteria remodels actin filaments (red) during entry and traffics along microtubules (green) to the perinuclear region. Inclusion membrane proteins (Incs) recruit the Golgi complex (yellow) and endoplasmic reticulum (blue). At the end of the intracellular cycle, the inclusion exits via actin-dependent extrusion or cell lysis. ( **b**) Anatomy of the female genital tract and epithelial cell organization in the lower and upper tract. ( **c**) New epithelial model systems (left) and schematic of polarized columnar epithelial cell–cell junctions (right). Tight junction (TJ) and adherens junction (AJ) complexes recruit adaptor proteins that connect to the actin cytoskeleton (red); desmosomes interact with intermediate filaments (yellow). ( **d**) New genetic tools for *C. trachomatis* include chemical mutagenesis and whole-genome sequencing to identify mutations and plasmid transformation to generate fluorescent reporter strains, tagged effectors, and targeted gene disruption via allelic replacement. ( **e**) New proteomic-based strategies to identify host proteins that interact with *Chlamydia* Incs. Incs tagged with the enzyme ascorbate peroxidase (APEX) (left) can ligate biotin-phenol on host proteins in close proximity. Purified inclusions (middle) and Strep-tagged Incs (right) were used to identify host proteins recruited to the inclusion and Inc–host protein interactions, respectively. ( **f**) Summary of advanced microscopy approaches to visualize *Chlamydia* effector localization using the Split-green fluorescent protein (Split-GFP) system (left), the structure of the T3S apparatus in contact with the plasma membrane (middle), and reticulate body (RB)-to-EB conversion (right). ZO-1, zona occludens 1.

All *Chlamydiae* encode a type III secretion (T3S) system to deliver a defined cohort of bacterial proteins (“T3S effectors”) directly into the host cell ^5^. The *Chlamydia trachomatis* EB T3S effectors modify the cytoskeleton and stimulate bacterial uptake into a membrane-bound vacuole that is rapidly segregated from degradative trafficking pathways ( Figure 1A) ^6, 7^. A subset of T3S effectors are inserted into the inclusion membrane. These inclusion membrane proteins (Incs), which are secreted throughout the infectious life cycle, are diverse (~5% of the total coding potential of *C. trachomatis*) and their molecular function is just beginning to be understood ^8^. For instance, Incs co-opt the microtubule motor protein dynein to transport nascent inclusions along microtubules toward the centrosome ( Figure 1A) ^9^. Along the way, the inclusion membrane is modified by lipid kinases, which may be important for evasion of endolysosomal compartments ^10^. Some Incs contain SNARE (soluble *N*-ethylmaleimide–sensitive factor attachment protein receptor)-like domains that coordinate fusion between inclusions and other membrane vesicles ^11^, whereas others promote the recruitment of the endoplasmic reticulum and Golgi complex to the vicinity of the inclusion, possibly to intercept lipid-rich vesicles to support *Chlamydia* replication ( Figure 1A) ^12– 14^. As the inclusion matures, it is increasingly encased by a network of F-actin, microtubules, intermediate filaments, and septins, which help confine the bacteria within the inclusion and limit recognition of bacterial products by innate immune sensors ^15– 17^.

Mid-stage through the infectious cycle, RBs transition back to EBs such that at the end of the cycle the infectious bacteria are released either by an actin-dependent extrusion process whereby the intact inclusion is exocytosed from the cell or by lysis of the host cell which requires the cleavage of cytoskeletal elements and nuclear rupture ^18, 19^.

Much of our understanding of the cell biology of how *Chlamydia* interacts with target cells and the molecular mechanisms it uses to manipulate cellular processes is based on observations made in infected cancer cell lines, which in addition to being metabolically and genetically adapted for proliferation, lack positional cues that are available only in the context of tissues. For example, polarized columnar epithelial cells, the *in vivo* target of *C. trachomatis*, display a spatial organization of organelles and cell signaling pathways that intimately intertwine stable cell–cell junctions to epithelial function and proliferation ( Figure 1B, C). Such structures and signaling networks are not present—or properly wired—in common cell lines used in *Chlamydia* research. Fortunately, more sophisticated infection models, coupled with the increasing ability to genetically manipulate *Chlamydia*, now provide a renewed tool kit to better understand how these pathogens interact with their intact animal hosts.

## An expansion of the molecular tool kits available for *Chlamydia* research

The greatest single advance in *Chlamydia* biology over the past decade has been the development of methods to perform genetic analysis of *C. trachomatis* mutants and increasingly robust molecular genetic tools to transform *Chlamydia* with recombinant DNA ( Figure 1D) ^20– 22^. As a result, it is now possible to perform targeted gene inactivation and plasmid-based complementation of chromosomal mutations ^22– 25^. The ability to express exogenous proteins, epitope tags, and fluorescent and other reporter proteins in *Chlamydia* has also expanded the repertoire of possible technologies that can be applied to study the *Chlamydia*–host interface ^26, 27^. For instance, Incs fused to enzymes that enable the biotinylation of proteins identified host factors that are in proximity to the inclusion membrane by affinity capture of biotinylated proteins coupled with tandem mass spectrometry ( Figure 1E) ^28, 29^.

Two recent studies using quantitative proteomics identified host proteins that interact with Incs and host proteins that are preferentially recruited to the inclusion ( Figure 1E). In one study, intact inclusions were isolated and all associated host proteins were identified and quantified by stable isotope labeling by/with amino acids in cell culture (SILAC)-based mass spectrometry ^30^. In parallel, a large-scale study identified previously unknown host-binding partners for nearly two thirds of the predicted Incs. Using affinity purification of Strep-tagged Incs coupled to quantitative mass spectroscopy, a second study provided a blueprint for Inc–host interactions ^31^. The different but complementary approaches presented new clues as to the potential mechanisms used by *Chlamydia* to subvert various aspects of host cell biology.

The advances in genetic and biochemical approaches have been further complemented with high-resolution microscopy ( Figure 1F). The localization and dynamics of *Chlamydia* T3S effectors can now be visualized live by using the split-green fluorescent protein (split-GFP) system ^32^, an approach that relies on fusing the effector with the 16–amino acid GFP-11 β-strand and infecting cells expressing the GFP1-10 β-strands. Upon secretion and complementation, the GFP β-barrel properly folds and fluoresces ( Figure 1F) ^33^. Furthermore, the application of cryo-electron microscopy revealed with unprecedented resolution that the *Chlamydia* T3S apparatus changes shape and polarizes toward host cell membrane ^34^. Similarly, applied serial block-face scanning electron microscopy temporally characterized the process of RB-to-EB conversion during the infection cycle ( Figure 1F) ^35^.

## Recent advances in cell culture infection models

Urogenital serovars of *C. trachomatis* target the mucosal epithelium. In the female genital tract, the infection typically begins in the endocervix before ascending to the endometrium and fallopian tubes ^36^. These tissues are comprised largely of polarized columnar epithelial cells with a rich diversity in form and function which is not readily recapitulated in two-dimensional culture settings ^37^. Thus, new *in vitro* models that more accurately reconstruct the organization and complexity of the genital tract are essential to better understand the full impact of *Chlamydia* infection on the epithelial physiology.

Wyrick and colleagues first described key differences in *Chlamydia* growth in polarized epithelial cells ^38^. Human endometrial epithelial cancer cells polarized on collagen-coated microcarrier beads significantly enhanced the growth of *C. trachomatis* serovar E, independent of EB attachment efficiency, compared with non-polarized cells ^38^. Infections in polarized enterocytes show that the inclusion preferentially captures lipid-rich exocytic vesicles that are specifically trafficked toward the basolateral membrane, suggesting that *Chlamydia* has adapted to grow in a polarized environment ^39^. More recently, a human endocervical epithelial cell line (A2EN cells) derived from a healthy patient sample has been shown to polarize, secrete mucin, and express pro-inflammatory cytokines during infection ^40, 41^.

Meyer and colleagues pioneered the use of *ex vivo* organotypic cultures and a novel fallopian tube organoid (FTO) model to investigate *C. trachomatis* infection in primary human epithelia ^42, 43^. Partially dissected tissue from the ectocervix and fallopian tube—representing the lower and upper genital tracts, respectively—has been cultured *ex vivo* and infected with *Chlamydia* ^42, 43^. These models were recently simplified by culturing isolated tubal epithelial cells in a three-dimensional matrix, generating self-renewing FTOs ( Figure 1C) ^44^. FTOs consist of secretory and ciliated epithelia, the two most common epithelial cell types in the fallopian tube, and more accurately recapitulate fallopian tube epithelial architecture. Acute infection in FTOs produced a strong inflammatory response while long-term and chronic infection increased epithelial stemness and proliferation and altered the methylation status of genes associated with aging. The latter results provide new insights into the long-term effects of *Chlamydia* infection on epithelial tissue homeostasis and the correlation between infection and cell proliferation ^45^.

## The epithelium of the female genital tract

Ultrastructural analysis of the lower female genital tract shows squamous ectocervical epithelia and columnar endocervical epithelia, which display differential organization of cell–cell junctions ^37^. The endometrium resembles columnar epithelia of the endocervix with increased epithelial diversity that includes ciliated and secretory cells and hormonally responsive and crypt-like glandular epithelia ( Figure 1B) ^46^.

Ultimately, the protective function of the epithelium is accomplished through the formation of intercellular junctions, molecular complexes that link up with the cytoskeleton to reinforce epithelial integrity. However, unlike other epithelial tissues, the endometrium exhibits remarkable changes during hormonal cycles and pregnancy, directly altering the expression profile of cell–cell junction proteins, their organization, dynamics, and barrier function ^46, 47^. In columnar epithelia, the three main classes of intercellular junctions are the tight junctions (TJs), adherens junctions (AJs), and desmosomes ( Figure 1C) ^48^. Although their function as molecular linkages has long been appreciated, newer studies have uncovered critical roles in transducing cell signaling pathways during tissue damage, repair, and pathogenic infection ^49^.

At the apex of the lateral cell membrane, TJs maintain a fence that physically separates the apical and basolateral membranes while gating the flux of ions and solutes through the paracellular space ^50^. Three families of transmembrane proteins—claudins, occludins, and junctional adhesion molecules (JAMs)—form homotypic interactions between adjacent cells. Adaptor proteins—that is, zona occludens 1–3 (ZO-1–3)—bind to their cytoplasmic tails and scaffold the recruitment of the polarity complex, which specifies apical membrane identity, and actin and microtubules to regulate TJ dynamics and stability ^50^.

AJs along the basolateral membrane are formed by homotypic interaction between cadherins, calcium-dependent transmembrane proteins ^51^. The adaptor proteins α- and β-catenin bind to cadherins and the actin cytoskeleton to reinforce AJ stability ^52^. AJ components, including β-catenin and others such as YES-associated protein (YAP), also function as transcription factors but are excluded from the nucleus through their association with stable AJs ^53^. AJ assembly is initially mediated by nectins, calcium-independent adhesion molecules, that also bind to AJ and TJ adaptor proteins ^54, 55^. The related but structurally distinct desmosomes are composed of a second class of cadherins—desmogleins and desmocollins—that connect to intermediate filaments, such as keratins, rigid cytoskeletal elements that provide additional structural support. Indeed, these strong junctions are thought to resist mechanical stress and are essential for the maintenance of epithelial integrity ^56^.

Many pathogenic viruses and bacteria have evolved diverse strategies to subvert cell–cell adhesions to gain entry into host cells or penetrate further into the underlying tissue ^57^. For example, *Listeria monocytogenes* surface protein InlA binds to E-cadherin and the hepatitis C virus binds to claudin-1 to promote their internalization ^58, 59^. The enteropathogenic *Escherichia coli* T3S effector EspF disrupts TJs and leads to a loss of epithelial barrier function ^60^. Importantly, these perturbations to cell–cell junctions can elicit an immune response as lumenal bacteria and toxins leak into the underlying tissue ^61^.

## *Chlamydia* interactions with the epithelial surface

Initial observations in tumor cell lines indicated that *C. trachomatis* infection can impact cell–cell junctions. *Chlamydia* infections disrupt N-cadherin–based AJs in HeLa cells. Gaps were observed between *Chlamydia*-infected cells and β-catenin relocalized to inclusions based on indirect immunofluorescence ^62^. These basic observations in two-dimensional cell culture were detailed further in an *ex vivo* fallopian tube infection model. Infection with a *C. trachomatis* lymphogranuloma venereum (LGV) biovar disrupted epithelial polarity and cell–cell junction organization through a Wnt-driven paracrine signaling pathway ^42^. The Wnt pathway consists of secreted glycoproteins that bind to the Frizzled and LRP5/6 receptors, activating downstream signals to regulate tissue organization through cell fate determination, polarity formation, and cell growth ^63^. Activation of the canonical pathway disrupts the “destruction complex”, stabilizing the Wnt effector β-catenin and allowing its translocation to the nucleus, where it activates target genes ^63^. In fallopian tube epithelia, β-catenin translocates to the inclusion and a component of the destruction complex, adenomatous polyposis coli (APC), shows more diffuse cytoplasmic localization. Notably, the localization of these Wnt effectors also changed in neighboring uninfected cells but was rescued upon the addition of Wnt inhibitors, suggesting that infection can alter tissue-level Wnt signaling programs ^42^. More recently, pharmacological inhibition of Wnt signaling in endometrial epithelial cells resulted in smaller and aberrant inclusions and significantly reduced the production of EBs ^64^.

*Chlamydia pneumoniae*, the human respiratory pathogen and causative agent of roughly 4 to 6% of community-acquired pneumonia ^65^, also targets effectors of the Wnt pathway. The *C. pneumoniae* Inc Cpn1027 interacts with and may recruit the Wnt effectors Caprin2 and glycogen synthesis kinase 3 (GSK3) to the inclusion ^66^. The *C. trachomatis* T3S effector TepP regulates GSK3β recruitment to nascent inclusions in endocervical epithelial cells ^10^, but it is unclear whether these effectors regulate Wnt in the appropriate tissue context. In endothelial cells, C *. pneumoniae* infection also promotes vascular endothelial (VE)-cadherin phosphorylation on Y658 ^67^, a residue that can control AJ organization and barrier function ^68^. The actin-binding protein EPS8, which is recruited to nascent inclusions during invasion and interacts with phosphorylated peptides derived from the T3S effector Tarp, also binds to VE-cadherin, alpha-catenin, and TJ components to control junction organization ^69– 72^. These results suggest that *Chlamydia* may secrete effectors that can target components of cell–cell junctions and cell signaling pathways that regulate junction organization.

*Chlamydia muridarum*, a mouse-adapted *Chlamydia*, also alters the composition of TJ proteins in mouse oviduct epithelial cells ^73^. Infection with *C. muridarum* decreases the expression of ZO-1, claudin-1/2, occludin, and JAM-1 while upregulating the expression of claudin-3 and claudin-4. Accordingly, *C. muridarum* infection alters transepithelial electrical resistance, indicating that the barrier function of TJs is compromised. These phenotypes were exacerbated by the absence of the Toll-like receptor 3 (TLR3), which initiates double-strand RNA (dsRNA)-dependent type I interferon responses ^74^. How TLR3 signaling is linked to TJ remodeling is unclear, but type III interferons were recently reported to strengthen epithelial barrier function during *Salmonella* infection ^75^.

Mechanistically, it remains to be determined whether reprogramming of cell signaling pathways or the removal of adhesion components and recruitment to the inclusion or both underlie the disruption of cell–cell junctions. Nevertheless, *Chlamydia* targeting of cell–cell junctions may drive certain aspects of its pathogenesis, including infertility and ectopic pregnancies. During estrus, the endometrium undergoes extensive paracrine-driven remodeling of cell–cell junctions to permit blastocyst adherence and invasion into the epithelial tissue ^46^. Thus, infection-mediated disruption of epithelial cell–cell junctions may impede implantation. Alternatively, *Chlamydia* infection is associated with elevated expression of the prokineticins receptor 2 (PROKR2), a G protein–coupled receptor that modulates TJ organization and paracellular permeability, which may prime tubal epithelia for implantation in fallopian tubes of patients with ectopic pregnancies ^76, 77^.

## *Chlamydia* activates epithelial-to-mesenchymal transitions

The remodeling of epithelial cell–cell junctions is a hallmark of both inflammation and cellular proliferation ^78^. New studies indicate that multiple *Chlamydia* biovars can induce the transformation of epithelial cells to a more mesenchymal-like state by activating the epithelial-to-mesenchymal transition (EMT) program. EMT is essential for organogenesis and tissue repair during which epithelial cells adopt features of cells in the mesenchyme like fibroblasts, altering their polarity and the organization of the actin cytoskeleton and cell–cell adhesions ^79^. Driven by growth factors (for example, transforming growth factor beta [TGF-β], hepatocyte growth factor, and epidermal growth factor) and hormones (estrogen), EMT can be aberrantly activated during tumorigenesis and inflammation-associated fibrosis ^78, 80^. Most notably, EMT is often coupled to the downregulation of E-cadherin, upregulation of cell–extracellular matrix (cell–ECM) adhesions, deposition of ECM proteins, and enhanced cell motility. This transition is thought to enable tumor cells to disseminate toward the lymph nodes and vasculature ^79^. Alternatively, inflammation-associated EMT is necessary for tissue repair and ceases at the end of the inflammatory response. However, inflammation during chronic infections can lead to sustained EMT, tissue damage, and organ fibrosis ^81, 82^.

*Chlamydia* infection can promote EMT in different epithelial subtypes and through multiple pathways. Oviduct epithelial cells infected with *C. trachomatis* serovar D increase the expression of microRNAs (miRNAs) that promote EMT ^83^. By immunofluorescence microscopy, infection downregulates the expression of E-cadherin while upregulating the EMT markers smooth muscle actin (SMA), the matrix-degrading enzyme matrix metalloproteinase-9 (MMP9), fibronectin, and the transcription factors SNAIL1/2 and ZEB1 ^83, 84^. Similar results were observed in conjunctival epithelial cells infected with *C. trachomatis* serovar B. Infection increases TGF-β expression which signals through SNAIL/ZEB2 and drives the downregulation of E-cadherin and upregulation of fibronectin and SMA. These changes may also involve epigenetic modifications as methylation of E-cadherin, fibronectin, and SMA genes were observed ^85^.

More recently, a global phosphoproteomic and transcriptomic analysis in ectocervical epithelial explants infected with *C. trachomatis* serovar L2 indicated that infection activates cell signaling pathways to promote an EMT-like signature ^43^. The infection initiates at the top of the stratified epithelia and progresses toward the basal layer, suggesting that altering cell–cell junctions during EMT may provide access for subsequent rounds of infection. Indeed, downstream of the MAPK pathway, the transcriptional factors ETS1 and ERF promote EMT in infected cells, leading to the downregulation of E-cadherin and increased cell motility and invasion ^43^. Collectively, these observations suggest that *Chlamydia* can promote the transformation of epithelial cells, which may contribute to cancers of the cervix and uterus. However, these results must be confirmed *in vivo* and in the proper tissue context.

## *Chlamydia* infection in new *in vivo* models

Extending these observations to a robust animal model of infection has remained challenging. Although the mouse-adapted *C. muridarum* is used extensively to study the host immune response in mice, this *Chlamydia* species is less genetically tractable than *C. trachomatis*. On the other hand, intravaginal inoculations of C57BL/6 mice with *C. trachomatis* often fail to induce significant pathology because the bacteria do not efficiently ascend to the upper genital tract ^86^. However, transcervical inoculations ^87^ that bypass the vaginal vault and the use of more permissive mouse strains (for example, C3H/HeJ) have improved the ability to monitor the infections in the mouse upper genital tract and ensuing pathology and infertility ^88^. *C. trachomatis* serovar L2 ascends to the upper genital tract in C3H/HeJ mice when inoculated intravaginally, stimulating a robust immune response and altering epithelial cell height ^89^. Future studies combining these new infection models and techniques with both host and bacterial genetics will promote better molecular dissection of *Chlamydia* pathogenesis in live animals.

## Future directions

The recent advances in the *Chlamydia* experimental tool kit have brought about a new era in *Chlamydia* research. With the ability to specifically disrupt genes and express genes *in trans*, new mechanisms by which *Chlamydia* subverts its host will be identified. Applying these molecular tools in model systems that better mimic the *in vivo* physiology will significantly accelerate our understanding of the infection process, especially as these tools are applied to other *C. trachomatis* serovars with distinct tissue tropisms. For example, using more sophisticated infection models and defined *C. trachomatis* mutants, we now test how specific virulence factors promote infection and affect epithelial cell growth, function, organization, and potentially transformation. However, more work is necessary to decode the genetics of the urogenital and ocular biovars and generate the relevant *in vivo* models of infection that best recapitulate the host responses observed in humans. Collectively, these new tools and models can be prioritized for the identification of new therapeutic targets or harnessed for the rational design of vaccines for this clinically important pathogen.
